# Multiple Maxillar Exostosis: Teaching point: Multiple buccal exostosis are rare but asymptomatic, unequivocal, and always benign conditions of the jaws

**DOI:** 10.5334/jbsr.1766

**Published:** 2019-04-04

**Authors:** Brice Dion, Bruno Coulier

**Affiliations:** 1 St Luc Bouge (Namur), BE; 2 Clinique Saint-Luc, Bouge, BE

**Keywords:** Buccal exostoses, exostoses, tori

## Case Report

**Figure 1:**
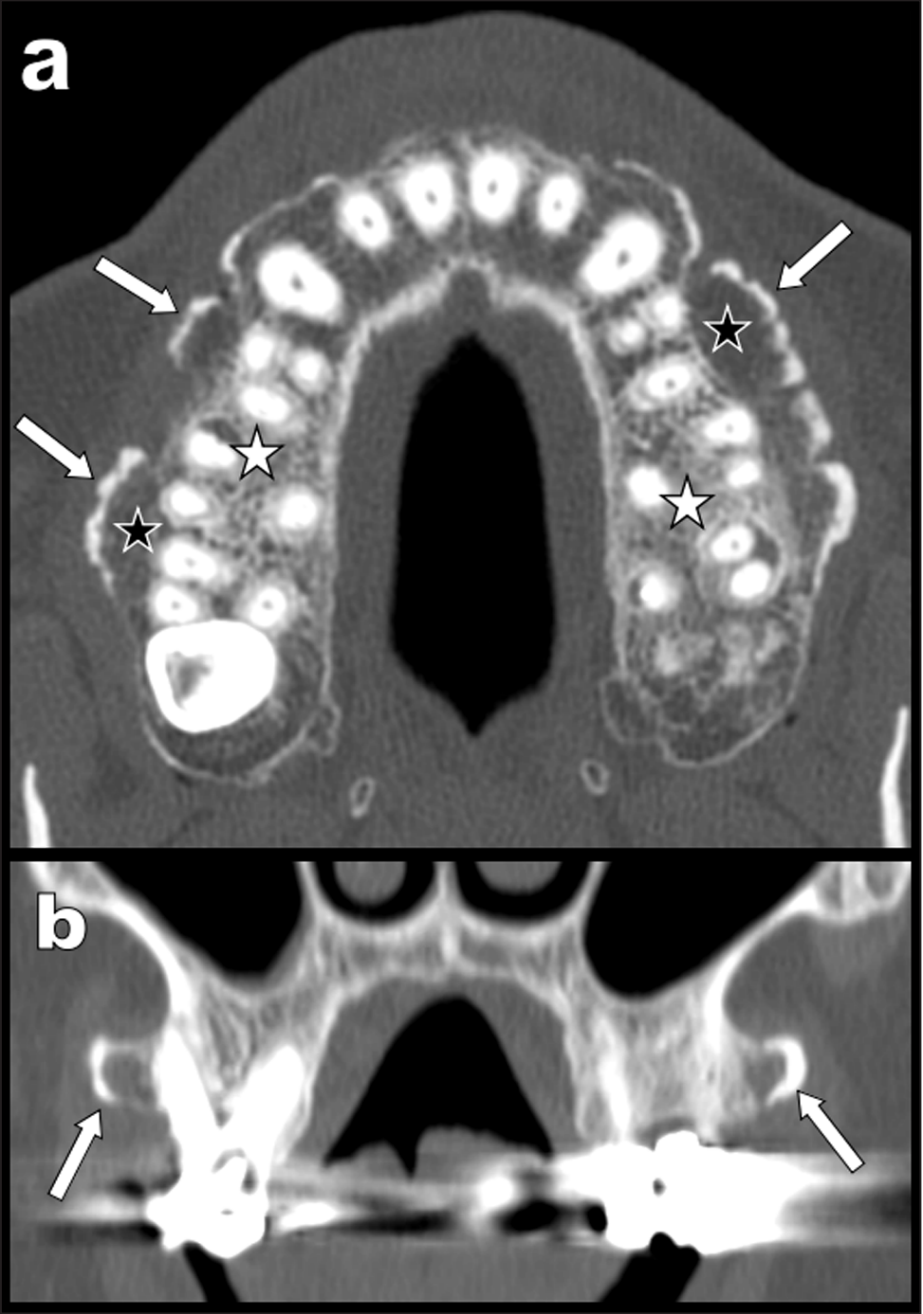


A 61-year-old man was referred for computed tomography (CT) of the sinusal cavities. Unusual lace-shaped bone growths were found all along the facial-vestibular side of the maxillary (Figure 1a). These growths had developed at the level of projection of apex of the dental roots. They appeared well-differentiated with a finely demarcated cortical bone (white arrows) covering a well-structured but rather loose cancellous bone (black stars). The density of this loose cancellous bone was sharply distinct from that of the more mechanically stressed cancellous bone (white stars) surrounding the dental roots.

**Figure 2:**
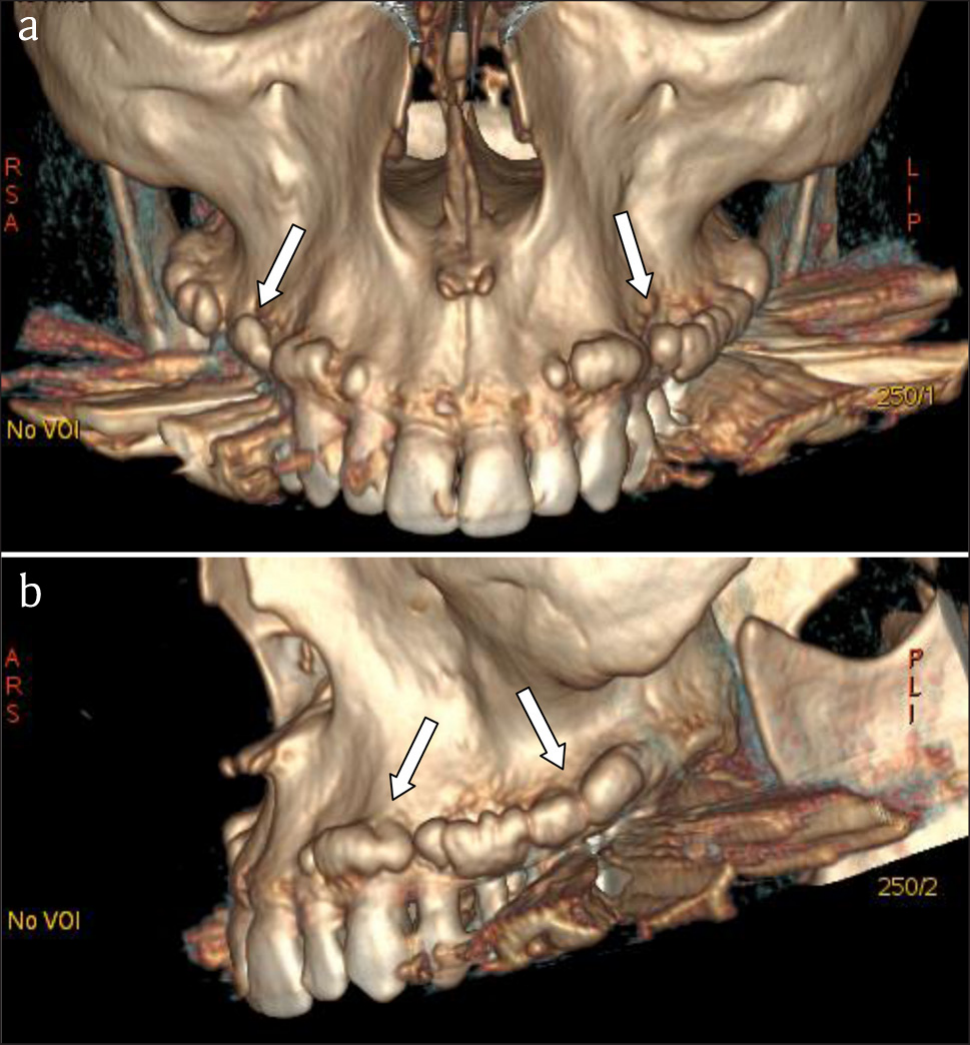


Coronal oblique reconstructions (Figure 1b) and bony volume rendering views (Figure 2a and b) illustrated the nodular appearance of these buccal exostoses (white arrows) giving to the global involvement a “pearl necklace” appearance.

These asymptomatic exostoses were known by the patient for a long time and had developed for many years. The patient had no mandibular exostosis, nor mandibular or palatine tori.

## Comment

Buccal exostoses are multiple and often bilateral benign bone protuberances arising from the maxilla and/or the mandibula. Their etiology remains unknown, though several factors might contribute to their development, such as genetic factors, increased masticatory function, or eating habits. Buccal exostoses might be of several types, usually classified according to their location [1]. The two most common types are commonly referred to as “tori”: The *torus palatinus* is a sessile, nodular bony mass commonly seen on the midline of the hard palate, and the *torus mandibularis* is a bony protuberance found on the lingual aspect of the mandible in the canine and premolar region. In contrast, vestibular maxillary and mandibular exostoses, arising from the facial aspect of the jaws, are considered much more rare.

Buccal exostoses typically develop during adolescence and keep growing slowly probably until the mid-thirties. Being mostly asymptomatic, mandibular exostoses are often found incidentally during dental care or during radiological investigations performed for other clinical reasons. There is no risk of malignant transformation, and thus no treatment is required in asymptomatic cases.

On radiographs, maxillar vestibular buccal exostoses appear as rounded opacities superimposed on the root of the teeth at the base of the maxillary sinuses. CT scans show nodular lesions made of well-differentiated normal bone with trabecular and cortical bone, arising from the facial aspect of the maxillary and/or mandibula.

Differential diagnoses include other benign bone tumors such as osteomas, ossifying fibromas, or fibrous dysplasia. The exclusion of a Gardner’s syndrome is recommended by some authors.
